# Fabrication of Highly Packed Plasmonic Nanolens Array Using Polymer Nanoimprinted Nanodots for an Enhanced Fluorescence Substrate

**DOI:** 10.3390/polym10060649

**Published:** 2018-06-10

**Authors:** Mohsin Ali Badshah, Jun Kim, Hoyoung Jang, Seok-min Kim

**Affiliations:** 1 Department of Mechanical Engineering, Chung-Ang University, Seoul 06974, Republic of Korea; mohsinali@cau.ac.kr (M.A.B.); zzangho@cau.ac.kr (H.J.); 2 Department of Mechanical System Engineering, Chung-Ang University, Seoul 06974, Republic of Korea; zuhn@cau.ac.kr

**Keywords:** polymer nanoimprinting, fluorescence, surface plasmon resonance

## Abstract

A simple and cost-effective fabrication method for plasmonic nanolens arrays (PNA) with a narrow gap has been proposed for fabricating enhanced fluorescence substrates, in which the fluorophores interacting with the enhanced electromagnetic field generated by localized surface plasmons provide a higher fluorescence signal. The PNA was fabricated by the sequential depositions of the SiO_2_ and Ag layers on a UV-nanoimprinted nanodot array with a pitch of 500 nm, a diameter of 250 nm, and a height of 100 nm. During the deposition processes, the shape of the nanodots changed to that of nanolenses, and the gap between the nanolenses was decreased via sidewall deposition. To examine the feasibility of the fabricated PNA for enhanced fluorescence application, a streptavidin-Cy5 (SA-Cy5) conjugate dissolved in a saline buffer solution was spotted on the PNA, and the fluorescence signals of the SA-Cy5 were measured and compared with those on a bare glass substrate. The enhancement factor was affected by the gap between the nanolenses, and the maximum enhancement factor of ~128 was obtained from the PNA with a SiO_2_ layer thickness of 150 nm and an Ag layer thickness of 100 nm. Finally, an electromagnetic field analysis was used to examine the fluorescence signal enhancement, and was conducted using rigorous coupled wave analysis.

## 1. Introduction

Fluorescence is a well-established detection method in DNA and protein microarray biosensor applications for achieving high throughput quantification of gene expressions or detecting antibody-antigen interactions [1,2,3]. Although a high correlation was reported between the results of traditional quantitative analysis methods and those of microarray biosensors at high molecular concentrations, the results of microarray biosensors at low molecular concentrations are limited and highly variable due to the low sensitivity of fluorescence-based detection [3,4]. Improving the sensitivity of fluorescence microarrays would be valuable for extending its use in clinical diagnostics and biological research. The surface plasmon resonance of metallic nanostructure, widely used for label-free detection [5,6], has been applied to enhance the fluorescence signal of the fluorophore, called metal-enhanced fluorescence (MEF). Metal-enhanced fluorescence (MEF) is a promising strategy for enhancing the sensitivity of fluorescence analysis. When an excitation laser light for fluorophore is irradiated on a metallic nanostructure, an enhanced electromagnetic (EM) field is generated around the nanostructure due to the localized surface plasmon resonance (LSPR) effect. The fluorophore located in the enhanced EM field of the metallic nanostructure absorbs more excitation energy and generates a higher fluorescence signal than the fluorophore on a bare glass substrate [7,8,9]. The fluorescence enhancement of the MEF substrate can be improved by increasing the confined EM at the gaps or sharp edges of nanostructures [10].

A number of approaches have been reported for fabricating the MEF substrates, which can be categorized as either naturally generated random nanostructure substrates [11,12,13] or engineered nanostructure substrates [14,15,16,17]. Although the naturally generated random nanostructures can be obtained by a simple fabrication process, the enhancement of the fluorescence signal is limited due to the limited shape controlling ability of the process. On the other hand, due to the ability to achieve the nanoscale precision in the design and fabrication processes, the engineered nanostructure substrate allows for the selection of geometrical variables (for example, shape, period, and size) to maximize the enhancement factor of the MEF substrates in a predictable way. An E-beam lithography (EBL) technique has been employed to fabricate uniform, closely packed, pillar and triangular nanostructure arrays; this is because a narrow gap or sharp edges are required for maximizing the MEF performance and EBL provides the smallest critical dimension [17]. However, the previous MEF substrate prepared by EBL had a small active MEF area (nano-patterned area, typically 36 μm^2^–0.4 mm^2^) [16,17] because of the time-consuming characteristic of EBL. To apply the MEF technique to the conventional DNA/protein microarray, a slide glass (25 × 75 mm^2^) MEF substrate fully covered by a metallic nanostructure is required. A well-known low-cost and large-area nanopatterning technique, nanoimprinting [18,19,20], can be utilized to fabricate a large-area engineered nanostructure MEF substrate. However, an expensive high-resolution patterning technique is still required in the nanoimprinting process to fabricate the initial master pattern.

In this manuscript, we propose a method to fabricate a large area via a highly-packed plasmonic nanolens array (PNA) using a nanoimprint and physical vapor deposition, in which a KrF laser scanning lithographed eight-inch silicon master equipped with a nanodot array with a diameter of 250 nm and pitch of 500 nm was used. A polymer nanodot array having similar dimensions to that of the master was fabricated by nanoimprinting on the whole area of the slide glass substrate, and SiO_2_ and Ag layers were sequentially deposited. Among Au and Ag, which are common materials for the MEF substrate, Ag was selected as a metallic material in this study due to its cost-effectiveness. Although the natural oxidation of Ag might deteriorate the fluorescence enhancement, the oxidation thickness is just a few nanometers and it does not cause a big degradation to enhanced EM field [21]. During the deposition process, the dot shape is changed to a lens shape and the gap between the nanolenses was decreased via the side wall deposition effect. To examine the feasibility of the proposed MEF substrate fabrication method, the effect of the deposition thickness on the fluorescence enhancement factor (FEF) was experimentally examined and confirmed by rigorous coupled wave analysis (RCWA).

## 2. Experimental Methods

### 2.1. Fabrication of Plasmonic Nanolens Array

**Figure 1:**
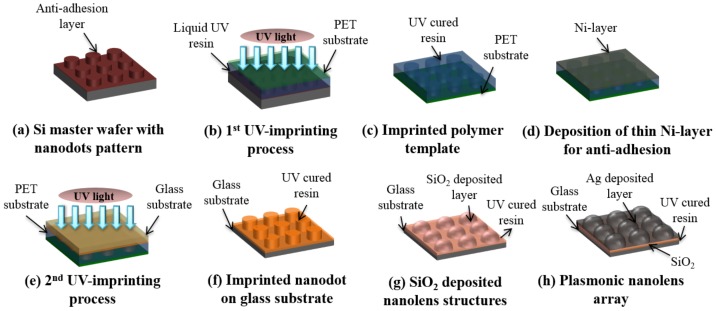
A schematic diagram of the plasmonic nanolens array fabrication using the nanoimprinting method.

A PNA was fabricated on a 25 × 75 mm^2^ slide glass substrate by a UV-nanoimprinting and physical vapor deposition process, as shown in Figure 1. An eight-inch silicon master containing the nanodot array was fabricated via KrF laser scanning photolithography (NSR-S203B, Nikon Co., Ltd., Tokyo, Japan) and reactive ion etching process [18]. The designed diameter, pitch, and height for the nanodot array master were 250, 500, and 100 nm, respectively. A self-assembled monolayer was applied onto the silicon master as an anti-adhesion layer by dipping the silicon master in a 2% solution of dimethyldichlorosilane dissolved in octamethylcyclotetrasiloxane (Repel-Silane ES, GE Healthcare Co., Ltd., Chicago, IL, USA) to prevent adhesion during the following UV-nanoimprinting [18,22]. To fabricate a polymer nanodot array on a slide glass substrate, we applied UV-nanoimprinting twice. First, a replicated polymer template with nanohole structures was obtained from a silicon master by utilizing a UV-imprinting process on a primer-treated polyester (PET) film (SH34, SKC Co., Ltd., Seoul, Korea) using a UV curable urethane acrylate-based photopolymer (UP088, SK Chemicals Co., Ltd., Seongnam, Korea) (Figure 1a–c). To use the first UV-nanoimprinted polymer film as a template in the second UV-imprinting process, a nickel (Ni) layer with a thickness of 10 nm was deposited onto the polymer template using an e-beam evaporator (Modified SEE-7, Ultech Co., Ltd., Daegu, Korea) (Figure 1d). This thin nickel layer could provide an anti-adhesion characteristic between the same UV-curable materials. The second UV-nanoimprinting process was conducted on a slide glass substrate (25 × 75 mm^2^) using the Ni-coated polymer template, and a UV-nanoimprinted nanodot array was formed on the slide glass substrate (Figure 1e,f). In order to generate the nanolens shape structures, the SiO_2_ layers of various thicknesses were deposited using an E-beam evaporator (Figure 1g). During the deposition process, the nanodot structures evolved into nanolens-shaped structures via sidewall deposition. Finally, a Ag layer with a thickness of 100 nm was deposited to obtain a PNA MEF substrate. Both SiO_2_ and Ag layers were deposited at a rate of 0.3 Ả/s under the vacuum condition of 6 × 10^−6^ Torr. To evaluate the effects of gap distance between plasmonic nanolenses on the FEF, the PNA MEF substrate with various SiO_2_ layer thicknesses (50, 100, 150, and 200 nm) were prepared. In the microarray biosensor analysis using a microarray scanner, an optically thick metallic surface was prepared to increase the fluorescence signal enhancement, in which the emitted fluorescence light toward the substrate was reflected to the objective lens of the detection system. In this study, the Ag layer thickness was fixed with a 100 nm because the 100 nm Ag layer was optically thick and its plasmonic property was similar to the thicker Ag layer (150 and 200 nm), as shown in Figure S1b in the Supplementary Material.

### 2.2. Evaluation of Geometrical Characteristics

**Figure 2:**
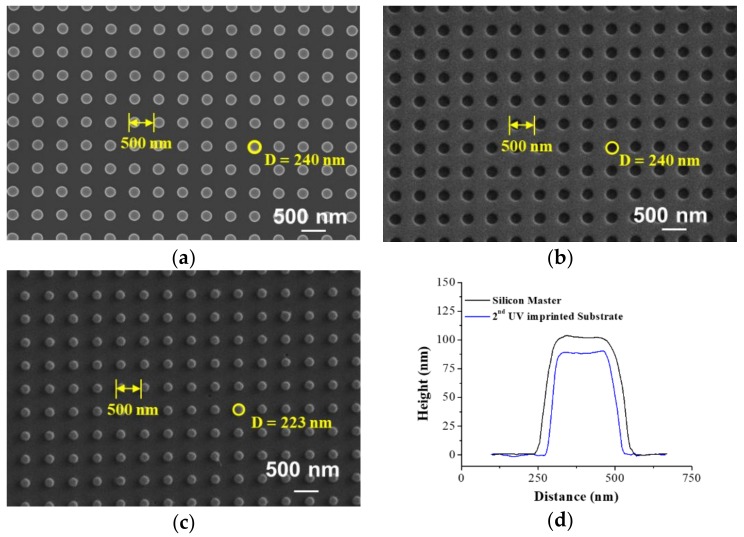
The top-view scanning electron microscope (SEM) images of (**a**) silicon master, (**b**) polymer master, (**c**) replicated nanopillar pattern, and (**d**) the comparison of the cross-sectional surface profiles of the silicon master and replicated nanopillar structures on a glass substrate obtained via atomic force microscope (AFM) measured results.

**Figure 3:**
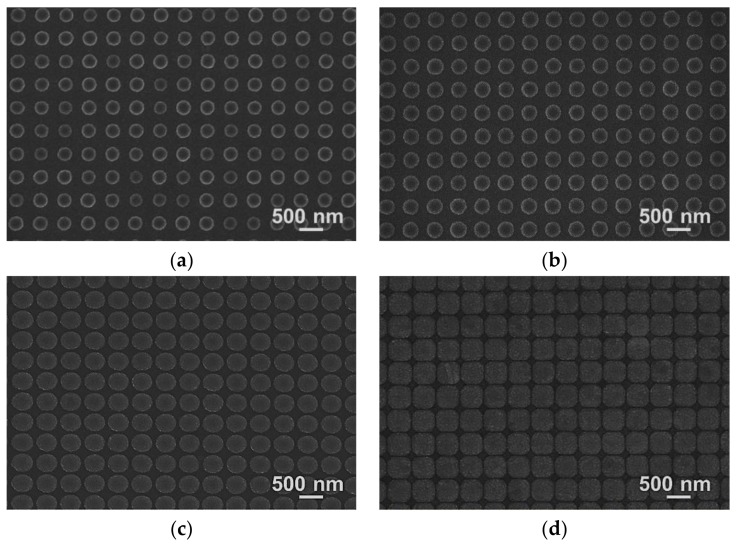
The SEM images of Ag nanolens array substrate with (**a**) SiO_2_ 50, Ag 100 nm, (**b**) SiO_2_ 100, Ag 100 nm, (**c**) SiO_2_ 150, Ag 100 nm, and (**d**) SiO_2_ 200, Ag 100 nm.

Figure 2a–c shows the top-view scanning electron microscope (SEM) images of the silicon nanodot master, first the UV-nanoimprinted polymer nanohole template and second the UV-nanoimprinted nanodot array on the slide-glass substrate. The measured diameters and pitches in the SEM images were 240 and 500 nm for silicon master, 240 and 500 nm for polymer template, and 233 and 500 nm for the nanoimprinted pattern, respectively. The slight difference between the designed diameter and measured diameter in the master pattern might have occurred during the reactive ion etching process. The dimensional change in the first polymer template fabrication process was negligible. However, the diameter of the final nanodot array was decreased due to the 10 nm Ni layer deposition process, where the Ni layer was used as an anti-adhesion layer on the polymer template. The dimensional change in the first polymer template fabrication process was negligible. However, the diameter of the final nanodot array was decreased due to the 10 nm Ni layer deposition process, where the Ni layer was used as an anti-adhesion layer on the polymer template. Figure 2d shows a comparison of the cross-sectional surface profiles of the nanodot pattern on the silicon master and nano-imprinted pattern on the slide glass obtained by an atomic force microscope (AFM) measurement. The XE-100 (Park Systems Co., Ltd., Suwon, Korea) was utilized for the AFM measurement using a non-contact AFM tip (PPP-NCHR-50, Park Systems Co., Ltd., Suwon, Korea) in the non-contact mode at a speed of 0.3 Hz. The measured height of the nanodots on the silicon master was 102 nm and that on the imprinted pattern was 91 nm. This deviation might be due to the polymer shrinkage during the first and second nano-imprinting processes. The PNA gap distance, which was the most important factor for determining the FEF of the PNA-MEF substrates, was controlled by the thickness of the deposited SiO_2_ layer. Figure 3 shows the top-view SEM images of the PNA with deposited SiO_2_ thicknesses of (a) 50, (b) 100, (c) 150, and (d) 200 nm, and a Ag thickness of 100 nm. It is clearly shown that the gap distance of PNA decreases and the diameter increases as the SiO_2_ layer thickness increases. When the thickness of the SiO_2_ layer exceeded 200 nm, the inter-lens spacing approaches zero, resulting in a square-shaped pattern that touches each other in the horizontal plane (Figure 3d).

**Figure 4:**
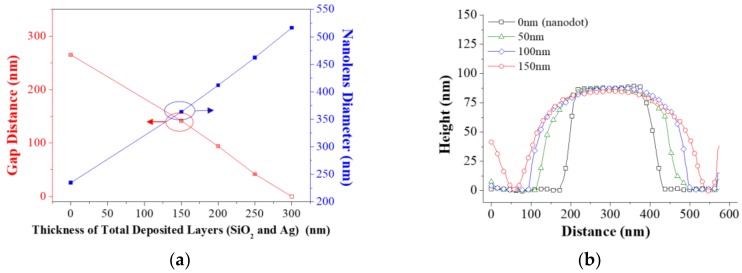
(**a**) The plot of the measured lens-to-lens spacing (left axis, red squares) and diameter (right axis, blue squares), and (**b**) measured AFM profiles of the nanolens structures as a function of the SiO_2_ layer thickness.

Figure 4a shows the effects of the SiO_2_ layer thickness with a fixed Ag layer deposition thickness of 100 nm on the gap distance and diameter of the PNA obtained by the SEM images. The PNA diameter at a 200 nm SiO_2_ layer thickness and 100 nm Ag layer thickness (total 300 nm) was measured in the diagonal direction due to the axial contact of adjacent nanolens in the horizontal direction. The increase of the PNA diameter according to the deposited layer thickness increase is almost linear with a rate of 1.25 nm/nm. Figure 4b shows the comparison of the cross-sectional surface profiles of PNAs with different SiO_2_ layer thicknesses and a 100 nm Ag-layer, as obtained by the AFM measurement results. It clearly shows that the initial nanodot pattern (black-dot) evolved to a lens shape as the SiO_2_ layer thickness increased.

### 2.3. Fluorescence Signal Measurements

To examine the FEF, the fluorescence intensities of a streptavidin-Cy5 conjugate (SA-Cy5, GE Healthcare Co., Ltd., Chicago, IL, USA) spotted on the PNA-MEF substrates were measured by a microarray scanner (GenePix 4000B, Molecular Device LLC, San Jose, CA, USA) with a laser wavelength of 635 nm which was the excitation wavelength for the fluorescence measurement. The SA-Cy5 was diluted in phosphate buffer silane (PBS, GE Healthcare Co., Ltd., USA) at different concentrations (100 ng/mL–100 μg/mL) was spotted on the PNA-MEF substrates and a glass reference substrate by pipetting with a volume of 0.4 μL. After a 24 h drying process, the fluorescence signals were measured and compared.

### 2.4. Electromagnetic Analysis 

To theoretically examine the fluorescence enhancement offered by the PNA MEF substrate, a rigorous coupled wavelength analysis (RCWA) was conducted using a commercial software package (R-soft, Diffractmode, Synopsys. Inc., Mountain View, CA, USA). A PNA structure was simplified as an ideal spherical lens shape with a measured base diameter and height, which was composed of UV-imprinted nanodots (*n* = 1.482) and deposited SiO_2_ (*n* = 1.46) and Ag layers (*n* = 0.056 + 4.29). In the RCWA simulation, a y-direction polarized light with a wavelength of 635 nm illuminated the PNA from the vertical direction, and one-unit volume was set as the simulation region for the periodic *x*- and *y*-direction boundary condition. Finally, an EM intensity distribution was simulated.

## 3. Results and Discussion

**Figure 5:**
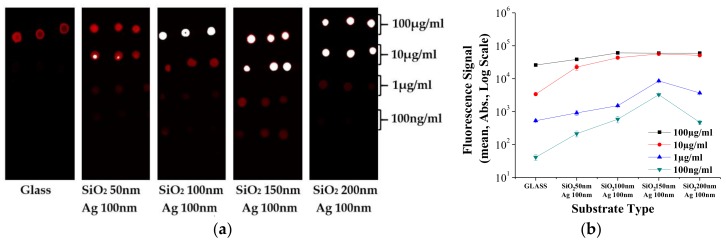
The comparison of measured fluorescence (**a**) microarray images and (**b**) intensity values at each streptavidin-Cy5 (SA-Cy5) concentration on the plasmonic nanolens array metal enhanced fluorescence (PNA-MEF) substrate and bare glass substrate (reference).

To examine the FEF of the PNA MEF substrate, the fluorescence signals of spotted SA-Cy5 at different concentrations (100, 10, 1, and 0.1 μg/mL) on PNA-MEF and bare glass substrates were measured using a microarray scanner, as shown in Figure 5. Figure 5a shows the microarray scanner images of the fluorescence spot for each concentration on the PNA-MEF substrate and glass substrate (reference). It was noted that the fluorescence signal of SA-Cy5 with concentrations lower than 10 μg/mL is difficult to see on the glass slide substrate, but were clearly observed on PNA-MEF substrates. For quantitative analysis, the mean fluorescence intensity was calculated in each spot and defined as the fluorescence signal. Figure 5b shows the comparison of the average fluoresce signal between each substrate type. The symbols represent the average values and the error bars denote the standard deviations of 27 spots (three spots per substrate and nine replicates/substrate). Since the fluorescence signals from 100 and 10 μg/mL SA-Cy5 were saturated on the PNA-MEF substrates, only 1 μg/mL and 100 ng/mL spot data were utilized for determining the FEF. The fluorescence signals from all PNA-MEF substrates were greater than that from the bare glass substrate. It was noted that the fluorescence signal from the PNA-MEF substrates increased with the increasing deposition thickness of SiO_2_ (decreasing gap distance) and were maximized at a SiO_2_ layer thickness of 150 nm. Since the same amount of SA-Cy5 was spotted on all substrates, the FEF was calculated by dividing the fluorescence signal of the PNA-MEF substrate into that of the glass substrate. The maximum FEF of ~128 was obtained from the PNA-MEF substrate with a SiO_2_ layer thickness of 150 nm.

**Figure 6:**
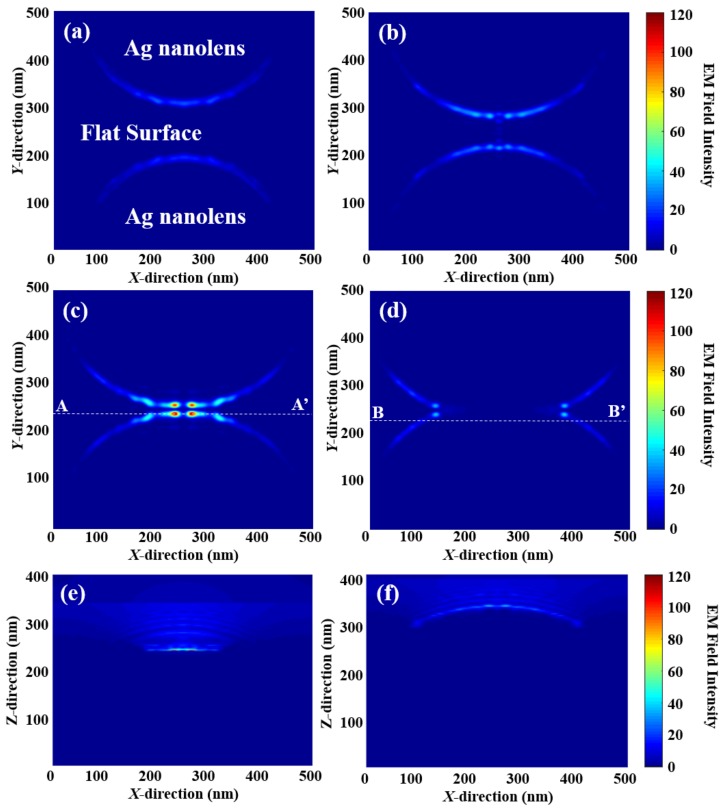
The RCWA simulation results of the EM field intensity distribution of PNA-MEF substrate with (**a**) 50, (**b**) 100, (**c**,**e**) 150, and (**d**,**f**) 200 nm SiO_2_ and 100 nm Ag layers; (**a**–**d**) *x*-*y* plane distribution at the PNA base plane and (**e**,**f**) the *x*-*z* plane distribution where the maximum EM field intensity was located; (**e**) the A-A’ line cross-section and (**f**) the B-B’ line cross-section.

To examine the correlation between FEF and enhanced EM intensity due to the LSPR, the 3-dimensional (3D) EM field intensity distribution of the PNA-MEF substrates was simulated using RCWA because the fluorescence enhancement is proportional to the square power of the EM field ${\left|E\right|}^{2}$ amplitude [23]. Figure 6 shows the RCWA simulated EM field intensity distribution for PNA-MEF substrate with (a) 50, (b) 100, (c,e) 150, and (d,f) 200 nm SiO_2_ and 100 nm Ag layers. The scale bar represents the enhancement levels of the ${\left|E\right|}^{2}$ distribution, normalized with respect to the incident EM-field (${\left|E_{inc}\right|}^{2}$) distribution. The Figure 6a–d shows the *x*-*y* plane EM field intensity distribution for the PNA-MEF substrates at the PNA base plane. In Figure 6a–c, an enhanced EM-fields was shown around the plasmonic nanolenses, and the maximum EM-field intensities in the whole 3D simulation results were found at the PNA base plane where the distance between the neighboring nanolens was minimized (near the *x* = 250 nm line). The maximum EM field intensity was achieved in the PNA with 150 nm SiO_2_ and 100 nm Ag layers (Figure 6c), which had the minimum gap distance. In Figure 6d, the maximum EM field at the PNA base plane was located at the contact edge of two nanolens because the two neighboring nanolens were overlapped. The maximum EM field intensity in the whole 3D simulation results of PNA with 200 nm SiO_2_ and 100 nm Ag layers was found in the *x*-*z* plane at *y* = 223 nm (B-B’ plane in (d)). Although the two neighboring nanolenses were overlapped in PNA with 200 nm SiO_2_ and 100 nm Ag layers as shown in Figure 3d, the EM field intensity at the interface was lower than that of PNA with a small gap in Figure 3c. This might be due to the overall shape change of PNA, with the increasing SiO_2_ layer thickness. With the increasing SiO_2_ layer thickness, the radius of the curvature of nanolens increases and the height of nanolens decreases after the overlapping. This means that the overall shape of PNA changed to the smoother surface which decreases the LSPR effects.

**Figure 7:**
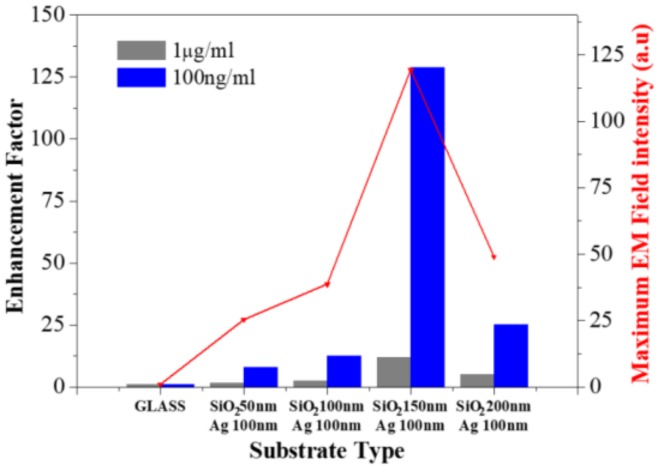
The effect of the SiO_2_ layer thickness on the fluorescence enhancement factor and maximum electric field intensity at 1 μg/mL and 100 ng/mL SA-Cy5 concentrations; the control signal was obtained from bare glass (reference).

Figure 7 shows the FEF and maximum EM field intensity of each PNA-MEF substrate. It was noted that the increase in the deposited-layer thickness decreases the inter-lens spacing, but generates higher LSP modes of resonance due to enhanced dipolar coupling, and enhances the EM field intensity [19,20]. Experimentally, we observed that FEF increased from 7.8× to 128.8× as the SiO_2_ layer thickness increased from 50 nm to 150 nm. Similarly, the EM field intensity increased from 25× to 119.4× with the decreasing inter-lens spacing 140 nm to 40 nm as a function of the SiO_2_ layer thickness. It was also noted that the FEF and EM field intensity decreased as the SiO_2_ layer thickness increased beyond 150 nm. The configuration of the nanolens shown in Figure 3d can suppress the dipolar coupling of the neighboring nano-objects, which leads to a decrease in the electric field intensity and the LSPR effect. This was further confirmed by the RCWA simulation, as shown in Figure 6d. The RCWA simulation shows excellent agreement with the experimentally measured FEF obtained during microarray imaging analysis. These results indicate that the PNA-MEF structures have a period of 500 nm with a spacing of 40 nm, obtained by depositing a SiO_2_ layer that is 150 nm thick and a Ag layer that is 100 nm thick, providing an opportunity for having optimum optical properties, which can be further applied to protein/DNA microarray analysis.

## 4. Conclusions

We have successfully demonstrated a MEF substrate consisting of a highly packed metallic plasmonic nanolens array fabricated utilizing a low-cost, large-area UV-nanoimprinting and evaporation process, in which the inter-lens spacing is precisely controlled through the thickness of the SiO_2_ and Ag layers subsequently deposited over a polymer nanodot array. An RCWA simulation was used to investigate the EM field distribution between adjacent nanolens; excellent agreement was observed between the experimentally measured FEF and simulated values for the EM field intensity. The inter-lens spacing was found as a critical factor for the enhancement of the fluorescence signal. A maximum enhancement factor of ~128 was obtained from the PNA-MEF substrate having a 150 nm SiO_2_ layer thickness with a spacing of ~40 nm. Based on the experimental and simulated results, we proposed that a PNA-MEF substrate fabricated by the proposed method is very attractive for the fluorescence-based sensing applications, because of its simplicity and cost-effectiveness. Although the plasmonic resonance wavelength of the fabricated PNA-MEF substrate was not exactly matched with the excitation wavelength of Cy5 as shown in Figure S1a in the Supplementary Material, the plasmonic resonance wavelength of PNA-MEF substrate can be tuned by changing the pitch of PNA, and a higher FEF can be obtained by matching the plasmonic resonance wavelength to the excitation wavelength.

## Supplementary Materials

The following are available online at http://www.mdpi.com/2073-4360/10/6/649/s1, Figure S1: Comparison of the simulated reflection spectra of PNA (a) varying the thickness of SiO_2_ layer (50~200 nm) with a fixed Ag layer of 100 nm, and (b) varying the thickness of Ag layer (50~200 nm) when the total thickness of SiO_2_ and Ag layers was fixed at 250 nm (narrow gap condition).
