# Supplementary material for: Fabrication of Highly Packed Plasmonic Nanolens Array Using Polymer Nanoimprinted Nanodots for an Enhanced Fluorescence Substrate
Source: Polymers (Basel). 2018 Jun 10;10(6):649. doi: 10.3390/polym10060649 (PMC6404152; doi:10.3390/polym10060649)
Supplement: Supplementary file 1 [file polymers-10-00649-s001.pdf]

polymers

MDPI

Supplementary Information

Fabrication of highly packed plasmonic nanolens array using polymer nanoimprinted nanodots for an enhanced fluorescence substrate

Mohsin Ali Badshah[1], Jun Kim[2], Hoyoung Jang[1], and Seok-min Kim[1,2,*]

[1]Department of Mechanical Engineering, Chung-Ang University, Seoul 06974, Republic of Korea; mohsinali@cau.ac.kr (M.A.B); zzangho@cau.ac.kr (H.J).
[2]Department of Mechanical System Engineering, Chung-Ang University, Seoul 06974, Republic of Korea; zuhn@cau.ac.kr (J.K).
*Correspondence: smkim@cau.ac.kr; Tel.: +82-2-820-5877

A1. Plasmonic property of fabricated PNA

The plasmonic property of the PNA was analyzed using the simulated reflection spectrum obtained by RCWA. Figure S1a shows the simulated reflection spectra of the PNAs with various SiO_2 layer thicknesses. Although the plasmonic resonance wavelength was not exactly matched to the excitation wavelength (λ = 635 nm), the fluorescence enhancement effect due to the LSPR of PNA could be obtained, because of the broad resonance characteristic of metallic nanostructure. The plasmonic resonance wavelength (the wavelength at the lowest reflectance) was red-shifted (move close to excitation wavelength) as increasing the thickness of SiO_2 up to 150 nm, and slightly blue-shifted at SiO_2 thickness of 200 nm, which was might be a reason for the maximum fluorescence enhancement at SiO_2 thickness of 150 nm. In addition, the reflectance at the excitation wavelength was minimized at the SiO_2 thickness of 150 nm. Since the plasmonic resonance wavelength can be tuned by changing the pitch of PNA, one can improve the fluorescence enhancement by matching the resonance wavelength to the excitation wavelength.

To examine the effects of Ag thickness on the plasmonic property, the reflection spectra of PNA with various Ag thicknesses were compared as shown in Figure S1b. To eliminate the structural effect, the total thickness of SiO_2 and Ag layers was fixed at 250 nm, which was the narrow gap condition in our experiment. The differences of simulated reflection spectra of PNA with Ag layer thickness of 100, 150 and 200nm were negligible. The reflectance of PNA with 50 nm Ag layer was lower than the others because the 50 nm Ag layer was not optically thick (partially transparent). However, the plasmonic resonance wavelengths of PNA with 4 different Ag thicknesses was exactly same. It means the plasmonic property was not sensitively affected by Ag layer thickness in the PNA MEF substrate. In this paper, we selected Ag layer thickness of 100 nm because the 100 nm Ag layer was optically thick and its plasmonic property was similar to the thicker Ag layer (150 and 200 nm).

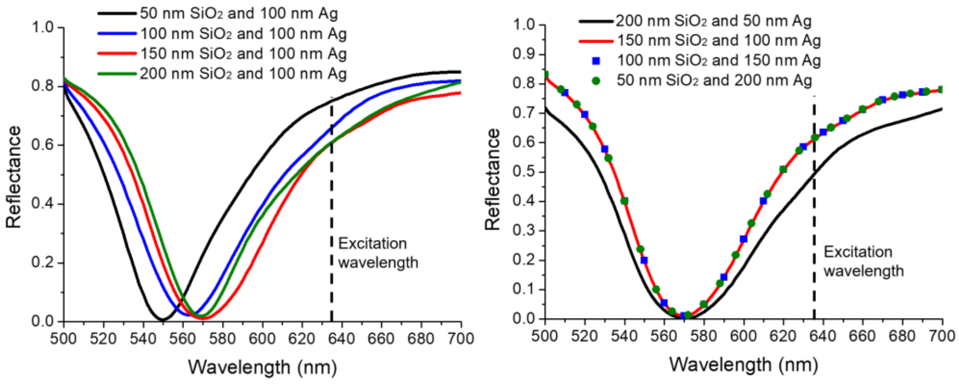

(a) (b)

Figure S1. Comparison of the simulated reflection spectra of PNA (a) varying the thickness of SiO_2 layer (50 ~ 200 nm) with a fixed Ag layer of 100 nm, and (b) varying the thickness of Ag layer (50 ~ 200 nm) when the total thickness of SiO_2 and Ag layers was fixed at 250 nm (narrow gap condition).
